# Quality Assurance of Beam Accuracy for Leksell Gamma Unit

**DOI:** 10.1120/jacmp.v1i1.2652

**Published:** 2000-01-01

**Authors:** Cheng Yu, Zbigniew Petrovich, Gary Luxton

**Affiliations:** ^1^ Department of Radiation Oncology University of Southern California, School of Medicine 1441 Eastlake Avenue Los Angeles California 90033; ^2^ Department of Radiation Oncology Stanford University School of Medicine Stanford California 94305

**Keywords:** beam accuracy, film dosimetry, Gamma Knife radiosurgery

## Abstract

For the acceptance test and annual quality assurance of the Leksell Gamma Unit, measurement of the beam accuracy, defined as a distance between mechanical and radiological isocenters, poses a challenge to medical physicists. The specification for the beam accuracy is within 0.5 mm for the 4‐mm collimator helmet. In this report, we introduce a simple technique to analyze the beam accuracy by using a conventional film densitometer plus mathematical modeling. A small piece of film was placed inside the film cassette containing a sharp needle. The needle is located such that its tip is exactly positioned at the mechanical isocenter. Before exposure, the film was pierced by the needle. Density profile was measured by using a densitometer with a spatial resolution of 0.8 mm. The profile was then fitted to a model of the two Gaussian functions. One is for the radiation field profile, the other for a dip caused by the narrow hole. The difference between the centers of the two Gaussian functions defines the deviation of the beam accuracy from the mechanical center of the unit. The deviations for *x, y,* and *z* directions from one of our annual measurements are 0.032, 0.054, and 0.195 mm, respectively. The combined deviation is 0.20 mm, which is well within the specification and in excellent agreement with the results from the manufacture's laser measurement. This technique provides a simple, accurate and practical tool for measurement of the beam accuracy in the acceptance test and annual quality assurance of the Leksell Gamma Unit.

PACS number(s): 87.66.–a, 87.53.–j

## INTRODUCTION

In stereotactic radiosurgery, such as Leksell Gamma Knife radiosurgery, a single high dose of radiation is delivered to a radiological lesion by using multiple high accurately focused beams.^1^ ^–^ ^3^ The accuracy of the radiation beam directly affects the accuracy of the patient treatment. For the Leksell Gamma Unit, the beam accuracy is defined as a distance or deviation between mechanical and radiological isocenters. During an acceptance test and/or annual quality assurance of the Leksell Gamma Unit, one of measurements is to verify the beam accuracy of the gamma unit.^4^ ^–^ ^8^ This measurement checks a deviation between mechanical and radiological isocenters. The manufacturer's (Elekta) specification for the beam accuracy is within 0.5‐mm for the 4‐mm collimator helmet (Leksell Gamma Unit User's Manual Vol. 2, Elekta, Norcross, GA). In this paper, we introduce a simple technique to analyze the beam accuracy of the Leksell Gamma Unit (Model U) developed at USC University Hospital by using a conventional film densitometer plus mathematical modeling.

## METHODS

The beam accuracy measurement was taken by using an accurately machined testing tool provided by the manufacturer. The testing tool consists of a rigid aluminum bar with a light‐tight film cassette at the middle of the bar. Inside the film cassette is a sharp needle, which is located such that its tip is exactly positioned at the mechanical isocenter of the gamma unit. The testing tool can be fixed to a collimator helmet by a pair of trunnions, which also define the *x* axis for the Leksell stereotactic head system. A small piece of film (Kodak, *XV* film) was carefully cut and placed inside the film cassette in a dark room. Before the film being exposed to radiation, the needle was pushed once to generate a small hole at the center of the film. After the exposed film was processed, the small hole at the center of the film was slightly enlarged by using a larger sharp needle for a later scan. The location of the hole pierced through a small piece of film is compared with the location of the center of the optical density distribution following exposure of the film. Density profile was measured by using a densitometer (Wellhofer WP102, Wellhofer, Germany) with a spatial resolution of 0.8 mm. The profile was then fitted to a model of the two Gaussian functions:

(1) $$\begin{array}{l} \text{Model}(x_{i},a,b,c,d,e,f)=a^{*}\exp\{-b^{*}{\left(x_{i}-c\right)}^{2}]+d*\exp[-e*{\left(x_{i}-f\right)}^{2}\}\\ \;i=1,2,\ldots ,n, \end{array}$$

where $x_{i}$ is a coordinate variable in the density profile, and *a, b, c, d, e,* and *f* are fitting parameters for the two Gaussian functions. The variable *n* ranges from approximately 150 to 250, depending on both width and speed of a particular scan. The first Gaussian function is for the radiation field profile, or the density profile, the other for a dip caused by the narrow hole. The mathematical fitting was achieved by using the “Minerr” function from commercially available software Mathacd 7.0 (MathSoft, Inc., Cambridge, MA). The “Minerr” function uses the Levenber‐Marquart method. This method requires a guess value for each unknown to begin the search for solutions. The mathematical difference between two centers (i.e., *c* and *f*) of the two Gaussian functions defines the deviation $\Delta_{i}(i=x,y,\text{or}\;z)$ of the beam accuracy from the mechanical center of the unit. By combining results from measurements in three perpendicular directions, the distance between the mechanically and the radiologically defined points are determined, i.e.,

(2) $$\Delta ={\left\{{\left(\Delta_{x}\right)}^{2}+{\left(\Delta_{y}\right)}^{2}+{\left(\Delta_{z}\right)}^{2}\right\}}^{1/2}.$$

## RESULTS AND DISCUSSIONS

**Figure 1:**
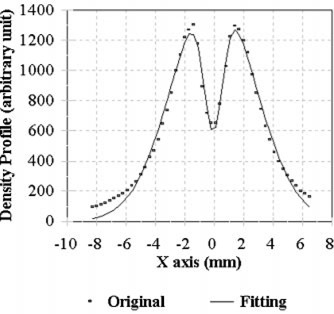
Determination of the beam accuracy for the 4‐mm collimator helmet. Dots represent the measured data of the density profile. Solid line is a fitted curve.

Figure 1 illustrates a result of the mathematical modeling of the *x* axis for the 4‐mm collimator from one of our annual quality assurance measurements. The zero in the scanning axis is arbitrary and just an origin of a scanning, not referring to either the mechanical or radiological centers. The deviations for *x, y,* and *z* directions from measurements were 0.032, 0.054, and 0.195 mm, respectively. The combined deviation was 0.20 mm, well within the specification and in excellent agreement with the results from the manufacturer's laser measurement.

The purpose of using a mathematical function to fit to the measured density profile is in an attempt to make best use of all measurement information. The other mathematical function may also be used for modeling.

Tables I and II show, respectively, measured relative coordinates and deviations of the mechanical and radiological centers for the 4‐mm and 8‐mm collimator helmets from one of the annual quality assurance measurements. The Elekta data for the 4‐mm collimator helmet is also included. These data were measured by the manufacturer for this gamma unit during the acceptance test in 1994. The signs (+ or −) in the second and third columns just reflect a coordinate relative to the scan origin.

**Table I:** Measured deviations for the 4‐mm collimator helmet.

	*C* ^a^ (mm)	*F* ^b^ (mm)	Deviation (mm)	Elekta data^c^ (mm)
*x*	0.043	0.075	0.032	0.025
*y*	0.007	‐0.047	0.054	0.075
*z*	0.158	0.355	0.195	0276
Combined deviation: 0.20 mm 0.29 mm				

a *C*–radiological center.

b *F*–mechanical center.

c From acceptance test made in 1994.

**Table II:** Measured deviations for the 8‐mm collimator helmet.

	*C* ^a^	*F* ^b^	Deviation (mm)	Elekta data^c^ (mm)
*x*	0.137	0.146	0.009	N/A
*y*	0.093	0.064	0.029	N/A
*z*	0.022	0.303	0.281	N/A
Combined deviation: 0.28 mm N/A				

a *C*–radiological center.

b *F*–mechanical center.

c From acceptance test made in 1994.

N/A–Not available

Beam accuracies for both the 4‐mm and 8‐mm collimator helmets for the six consecutive years are summarized in Table III. The average values are also listed and compared with Elekta's result. The measured data from this mathematical modeling technique are virtually identical to the values made by Elekta by using a laser film scanner.

**Table III:** Measured deviations from the last six years.

	Collimator	
Year	4 mm	8 mm
1994	0.29 mm	N/A
1995	0.26 mm	0.28 mm
1996	0.22 mm	0.24 mm
1997	0.23 mm	0.18 mm
1998	0.20 mm	0.28 mm
1999	0.29 mm	0.29 mm
Avg	0.25 mm	0.25 mm
Elekta	0.29 mm	N/A

N/A–Not available.

This technique provides a simple, accurate and practical tool for measurement of the beam accuracy in the acceptance test and annual quality assurance of the Leksell Gamma Unit.
